# Early Detection of Adverse Drug Reactions in Social Health Networks: A Natural Language Processing Pipeline for Signal Detection

**DOI:** 10.2196/11264

**Published:** 2019-06-03

**Authors:** Azadeh Nikfarjam, Julia D Ransohoff, Alison Callahan, Erik Jones, Brian Loew, Bernice Y Kwong, Kavita Y Sarin, Nigam H Shah

**Affiliations:** 1 Stanford Center for Biomedical Informatics Research Stanford Department of Medicine Stanford, CA United States; 2 Inspire Arlington, VA United States; 3 Department of Dermatology Stanford University School of Medicine Stanford, CA United States

**Keywords:** natural language processing, signal detection, adverse drug reactions, social media, drug-related side effects, medical oncology, antineoplastic agents, machine learning

## Abstract

**Background:**

Adverse drug reactions (ADRs) occur in nearly all patients on chemotherapy, causing morbidity and therapy disruptions. Detection of such ADRs is limited in clinical trials, which are underpowered to detect rare events. Early recognition of ADRs in the postmarketing phase could substantially reduce morbidity and decrease societal costs. Internet community health forums provide a mechanism for individuals to discuss real-time health concerns and can enable computational detection of ADRs.

**Objective:**

The goal of this study is to identify cutaneous ADR signals in social health networks and compare the frequency and timing of these ADRs to clinical reports in the literature.

**Methods:**

We present a natural language processing-based, ADR signal-generation pipeline based on patient posts on Internet social health networks. We identified user posts from the Inspire health forums related to two chemotherapy classes: erlotinib, an epidermal growth factor receptor inhibitor, and nivolumab and pembrolizumab, immune checkpoint inhibitors. We extracted mentions of ADRs from unstructured content of patient posts. We then performed population-level association analyses and time-to-detection analyses.

**Results:**

Our system detected cutaneous ADRs from patient reports with high precision (0.90) and at frequencies comparable to those documented in the literature but an average of 7 months ahead of their literature reporting. Known ADRs were associated with higher proportional reporting ratios compared to negative controls, demonstrating the robustness of our analyses. Our named entity recognition system achieved a 0.738 microaveraged *F*-measure in detecting ADR entities, not limited to cutaneous ADRs, in health forum posts. Additionally, we discovered the novel ADR of hypohidrosis reported by 23 patients in erlotinib-related posts; this ADR was absent from 15 years of literature on this medication and we recently reported the finding in a clinical oncology journal.

**Conclusions:**

Several hundred million patients report health concerns in social health networks, yet this information is markedly underutilized for pharmacosurveillance. We demonstrated the ability of a natural language processing-based signal-generation pipeline to accurately detect patient reports of ADRs months in advance of literature reporting and the robustness of statistical analyses to validate system detections. Our findings suggest the important contributions that social health network data can play in contributing to more comprehensive and timely pharmacovigilance.

## Introduction

Adverse drug reactions (ADRs) are an important public health issue, causing considerable patient harm with a high health care cost [1,2]. Serious ADRs occur in over 2 million patients annually in the United States, resulting in 100,000 deaths [2]. It is difficult to comprehensively characterize ADRs during premarket trials, as many serious ADRs, particularly rare ones, are discovered years after a drug has been on the market [3]. Clinical trials are limited in their ability to detect ADRs because of their relatively small cohort sizes, short durations, and a lack of enrolled patient diversity [4,5]. A significant number of ADRs are recognized in the postmarket period, resulting in over 2 million injuries and US $75 billion in annual health care costs [6,7]. Ongoing surveillance strategies are therefore necessary to monitor drug safety during the postapproval period.

Spontaneous reporting systems (SRSs) are drug safety surveillance mechanisms designed by regulatory agencies to monitor drug safety during the postmarket period. SRSs can effectively detect rare ADRs by nature of their longitudinal profiling and wide reach of included reports but are limited by their reliance on voluntary patient or provider reporting; it is estimated that more than 90% of ADRs are underreported [8,9]. ADR underdetection has motivated efforts to include complementary, alternative data sources for pharmacovigilance, including electronic health records and administrative claims [10-12], biomedical literature [13,14], Internet search logs [15], patient posts in social media [16-18], and multimodal systems that jointly analyze multiple sources of information for ADR detection [7].

Over 300 million patients seek and share health-related information from online Internet platforms such as health forums, search engine logs, or tweets [18]. These social health networks provide a platform for patients or caregivers to connect in discussing treatment options, drug side effects, and illness trajectories. The valuable health-related content of user posts in social media can be used for public health surveillance purposes [19], also termed *infodemiology* [20], including monitoring the spread of contagious diseases such as influenza [21,22]; monitoring the time and geographical locations of diseases [23]; health outcome measurement [24,25]; discovering associations between health-related concepts such as drugs and diseases; and, particularly, monitoring adverse effects of medications [16,17]. Several studies have highlighted the importance of utilizing social media as a resource for pharmacovigilance [9]. User posts often contain informal, unstructured text from which it is more challenging to extract medical information than from other, more-structured sources. Therefore, the exploration of different natural language processing techniques in ADR concept detection from social media postings has received significant attention from the medical informatics community [9,17], though there are a paucity of studies focusing on drug-ADR, signal-generation methods based on social media postings [26].

Here we use Inspire [27], one of the largest online social health networks, which contains over 12 million health-related patient posts, including discussions of therapy responses, adverse drug reactions, and supplemental treatments. We present an ADR signal-generation pipeline based on patient posts in social health networks and compare the timing and the rate of such ADRs with those published in clinical literature. We demonstrate the capacity for early detection as well as discovery of ADRs using Inspire content.

In this work, we focus on two classes of chemotherapeutics, targeted small molecule inhibitors and immunotherapeutics, which are representative of the dramatic change in the chemotherapy landscape since the early 2000s. These classes of agents are now commonly used in place of more traditional antiproliferative agents and are associated with novel side-effect profiles related to their mechanisms of action. Oncologists have experienced a particularly steep learning curve in recognizing these reactions, which occur in essentially all patients and can be life-threatening [4,28] as there is limited-to-no long-term data with novel agents. A new subfield of oncology has emerged, aimed at recognizing which reactions are reflective of treatment response, which warrant treatment cessation, and managing side effects to permit treatment tolerability. To capture the breadth of reactions seen, here we focus on two representative classes of cancer drugs: (1) epidermal growth factor receptor (EGFR) inhibitors, which are widely used by most oncologists for specific malignancies harboring EGFR mutations, have been in practice for over 15 years, and, therefore, have well-established side-effect profiles; and (2) immune checkpoint inhibitors, which are relatively new, having first gained US Food and Drug Administration (FDA) approval in late 2014 and, therefore, have significantly less data available on their emerging side-effect profiles. We report the construction of a pipeline to study the association of cutaneous ADRs with these selected targeted cancer therapy drugs reported in patient postings in Inspire.

## Methods

### Overview

We defined a set of common and rare ADRs to study for their association with two classes of drugs: an EGFR inhibitor, erlotinib, and the immune checkpoint programmed cell death 1 (PD-1) inhibitors, nivolumab and pembrolizumab. We focused on eight skin-related ADRs in this work: rash, acne, pruritus (ie, itchy skin), paronychia (ie, nail changes), xerosis (ie, dry skin), hypohidrosis, bullous eruption (ie, blister), and psoriasis.

### Drug Corpus Generation

The Inspire dataset consists of 7,320,546 discussion posts from 2005 to 2016. For each drug in the study, we generated a corpus defined as a collection of user posts from Inspire, with every post containing at least one mention of a keyword corresponding to one of the drugs of interest. To retrieve relevant posts from the Inspire dataset, we used regular expressions, a simple text-processing method for string matching. We created a regular expression pattern that implemented the exact string match. We have provided the list of the drugs and the related regular expression in Multimedia Appendix 1.

We identified 55,778 posts for erlotinib and 15,738 for the combined PD-1 inhibitors, nivolumab and pembrolizumab. Because nivolumab and pembrolizumab were more recently introduced and, therefore, have less discussion content but essentially identical mechanisms, we combined posts for the two drugs referencing the same ADR.

### Detection of Adverse Drug Reactions

To extract ADR mentions from user posts, we used DeepHealthMiner (DHM), a neural network-based named entity recognition (NER) system that is specifically trained to extract drug safety-related entities from user-generated content in social media [29]. DHM is a supervised feed-forward neural network that is trained to identify two different entity types: ADRs and drug indications. The original, labeled training data is based on a dataset from DailyStrength [30], an online health community that contains patient-generated reports of treatment experiences very similar to those from Inspire. The labeled training data are the sentences from patient posts manually annotated for the entity span (ie, start and end position offsets) and the type of entity (ie, ADR or indication). More detailed information about the data and annotation details can be found in prior publications [31,32]. The unlabeled sentences from user posts are also used for unsupervised training of the word embeddings, which are used as vectors representing the input tokens to the NER system.

We retrained DHM using both labeled and unlabeled posts from Inspire. We retrained 150-dimensional word embeddings by adding the 7 million Inspire posts to the original unlabeled sentences. We manually labeled an additional 200 Inspire posts, following the original annotation guideline [31,32], to retrain DHM using Inspire content. Some of the rare ADRs in this study are more frequently discussed as indication mentions (eg, psoriasis or blistering) rather than ADRs in user postings; therefore, to improve our system’s ability to correctly recognize such cases, we created a subset of 200 Inspire posts from our corpus that particularly contained keywords from the rare seed ADRs. We annotated all the ADR and indication mentions following the original annotation guidelines to retrain DHM using Inspire content. The preliminary results showed that the system could successfully extract mentions of rare ADRs after retraining on 200 posts.

To evaluate the performance of the retrained NER model in identifying ADR entities from Inspire posts, we created a test set—the Inspire test set—consisting of 50 randomly selected posts. Two human annotators labeled all mentions of ADRs, not limited to the selected ADRs in this study, corresponding to any treatment reported in the posts (interannotator agreement: 90.9%). We used the *F*-measure to quantify the interannotator agreement by considering one annotator as the gold standard [33].

The extracted ADR mentions along with the information about the related drugs were stored in a database for further analyses of the associations among drugs and ADRs.

### Normalization of Extracted Mentions

To map the extracted ADR mentions to Unified Medical Language System (UMLS) Concept Unique Identifiers (CUIs), we generated a lexicon of the eight ADR concepts. We defined a set of seed CUIs for these ADRs as shown in Multimedia Appendix 2. Using the UMLS hierarchy, we expanded every seed concept by adding synonyms and alternative names. For “nail changes,” we further expanded by including the children of the seed concepts (ie, is-a relation in the hierarchy). Every lexicon entry has a name, UMLS CUI, related seed ADR concept, and an identifier that we defined to group all items corresponding to a specific ADR. As an example, the lexicon entry “ingrown toenail (C0027343)” is an expanded concept based on “Disorder of nail (C0027339),” and “nail changes” is the human readable identifier for the ADR. Additionally, for every ADR, we added a set of colloquial phrases to the lexicon (see Multimedia Appendix 2). The list of colloquial phrases was generated by physicians, representing the list of words and phrases that they had seen patients use to describe their own cutaneous ADRs. We included these to better capture how patients describe ADRs in their own words.

Finally, we used Lucene [34] to index the lexicon. Lucene has been utilized as a successful tool in previous studies for normalizing biomedical entities [35,36]. We built a Lucene repository from the lexicon by creating one Lucene document for each lexicon entry, indexing the corresponding UMLS concept name and the CUI for each lexicon entry. The concept names are tokenized and lemmatized for generalization.

For mapping an extracted ADR mention to a UMLS concept, we searched the index using the text span extracted from the post by our NER system. We tokenized and lemmatized the extracted text span in the same manner as the indexed concept names and generated a Lucene query to search the index. For example, to map the extracted ADR mention “finger nails have been peeling” to a UMLS concept, we search the index using the query keywords “finger nail have be peel” and retrieve “peel nail (Peeling of nails: C0263531).” We retrieved a ranked list of the matched, relevant ADR concepts and chose the top-ranked concept from this list.

### Computing Drug and Adverse Drug Reaction Associations

The co-occurrence of a drug and a reported ADR in a post can be considered as a potential association. To quantify the strength of an association between a drug and an ADR, we calculated the proportional reporting ratio (PRR), a statistic widely used for ADR signal generation from spontaneous reporting databases [26,37] and originally introduced by Evans et al [29]. The PRR quantifies ADR signals by comparing the frequency at which an ADR is reported with a drug of interest compared to the frequency at which the ADR is reported with other drugs (ie, comparison drugs) in the database.

We defined the comparison drugs by sorting drugs mentioned in Inspire by discussion frequency and selecting those most highly discussed in Inspire discussion posts (frequency >5000). To reduce noise, we excluded over-the-counter drugs with common indications (eg, pain and allergy drugs), as well as several anti-inflammatory drugs (eg, prednisone and Humira) that have indications that match some of our rare ADRs (eg, bullous eruption and psoriasis), given that patients discussing these drugs were almost exclusively discussing their indications rather than experienced ADRs. The final comparison drug list included 27 drugs, which are listed in Multimedia Appendix 3.

The PRR for an adverse reaction, R, and a drug, D, was calculated based on the following formula:

PRR(D,R) = (count[D∩R] / count[D]) / (count[!D∩R] / count[!D])

We defined count(D∩R) as the number of unique users that have reported both D and R in a post; count(D) as the total number of users reporting any ADR for drug D, including all extracted mentions by DHM (eg, weight gain or fatigue); count(!D∩R) as the total number of unique users that reported R for comparison drugs, excluding D; and count(!D) as the total number of unique users that reported at least one ADR for any drug except D.

### Calibrating a Threshold for the Proportional Reporting Ratio

**Figure 1:**
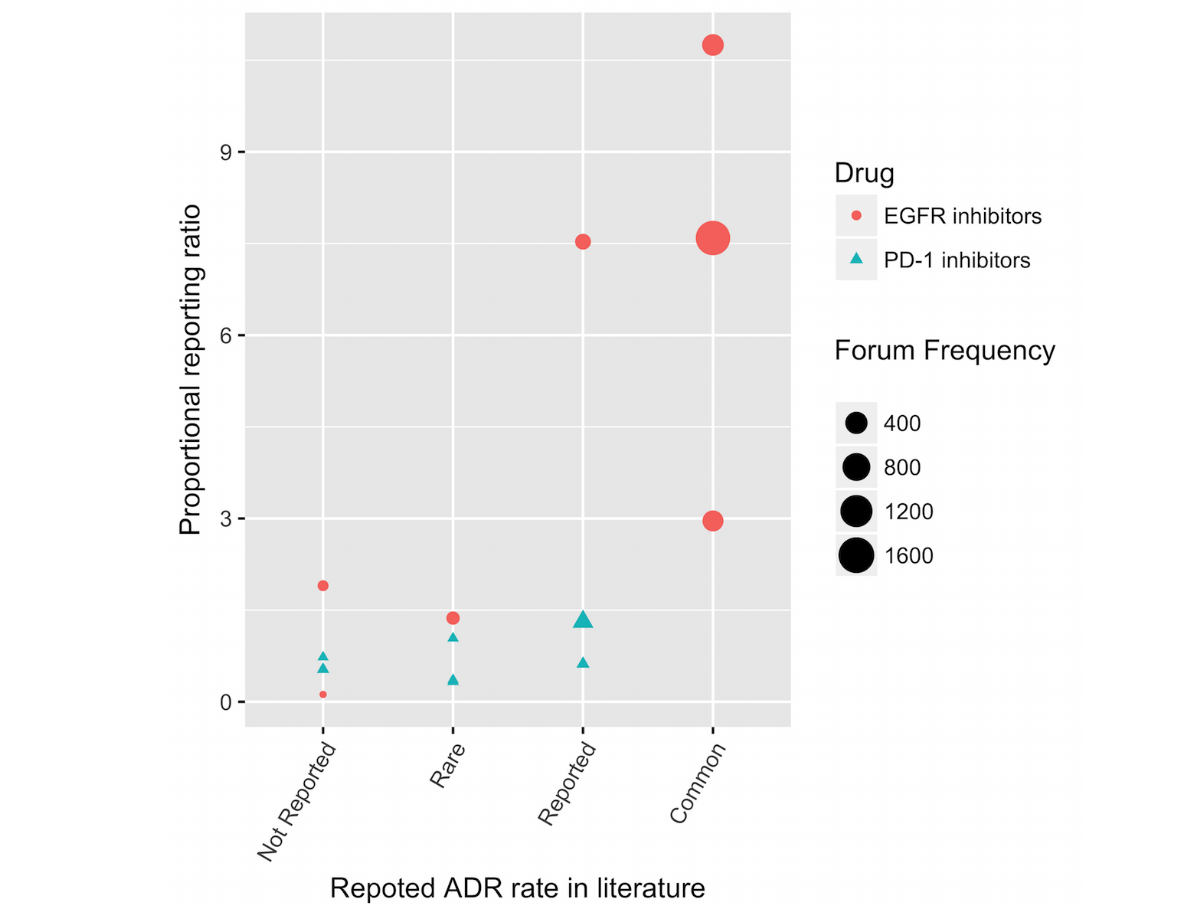
Comparison of proportional reporting ratio of skin adverse drug reactions (ADRs) reported in social health forums with the ADR rate published in the literature. The ADR rate based on the literature are grouped as follows: Not reported or rare = case report; reported = case series or in clinical trial; common = in significant percentage of patients in large trials. The frequency at which ADRs are talked about is shown by the size of the circle or triangle. The most common ADRs reported in the literature are also the most discussed in the patient posts. EGFR: epidermal growth factor receptor; PD-1: programmed cell death 1.

We performed empirical calibration to find the threshold at which the PRR may represent a true ADR signal [38,39]. We considered the distribution of PRR scores for a set of drug-condition pairs with no known associations. To create this negative control set, we first paired all 27 comparison drugs and the eight target ADRs, totaling 216 drug-condition pairs. We then excluded the drug-condition pairs with known associations (ie, ADR or indication) based on the Medi-Span Adverse Drug Effects Database (Wolters Kluwer Health) and side-effect resource (SIDER) [40], leaving 86 pairs without any documented associations. Based on the PRR formula, we expected and validated that drug-ADR pairs with known and common associations have PRR values greater than 1 (see Figure 1). If a drug-ADR pair has a known association, then the corresponding PRR will be relatively higher than that for pairs with no associations. Our goal in excluding these known pairs from the negative control set was to illustrate the distribution of PRRs for drug-ADR pairs with no known associations and use only these to calibrate the threshold at which a PRR for an unknown drug-ADR pair would represent a significant, novel result.

It is important to note that a drug and a condition can be associated for reasons other than an ADR or indication and may, therefore, co-occur frequently within the negative control set. We identified two common reasons that a drug (*drug* x) and a condition (*condition* y) with no documented association co-occur frequently in user posts, therefore representing false positives. The first reason is related to prescribing patterns: if a drug or a set of similar drugs are commonly prescribed with *drug* x, while the reported ADR condition is not attributed to *drug* x, the ADR will appear more frequently with all coprescribed drugs. For example, mentions of nail changes happen frequently with zoledronic acid; however, while a true ADR is associated with capecitabine, which is frequently prescribed with zoledronic acid, zoledronic acid is not responsible for the ADR. The second reason is that if *condition* y is one of the syndromic conditions related to the indication for *drug* x, the related condition will co-occur with the drug at a similar rate to the true indication. For example, metformin can be prescribed for polycystic ovary syndrome (PCOS), and patients with PCOS often have acne. Acne is not an ADR associated with metformin but will co-occur frequently with mention of metformin by way of acne’s association with PCOS.

Because of the potential for observing such drug-condition associations, we manually reviewed the negative control set to exclude them, leaving 81 drug-ADR pairs with no association. The negative control set is available in Multimedia Appendix 4.

**Figure 2:**
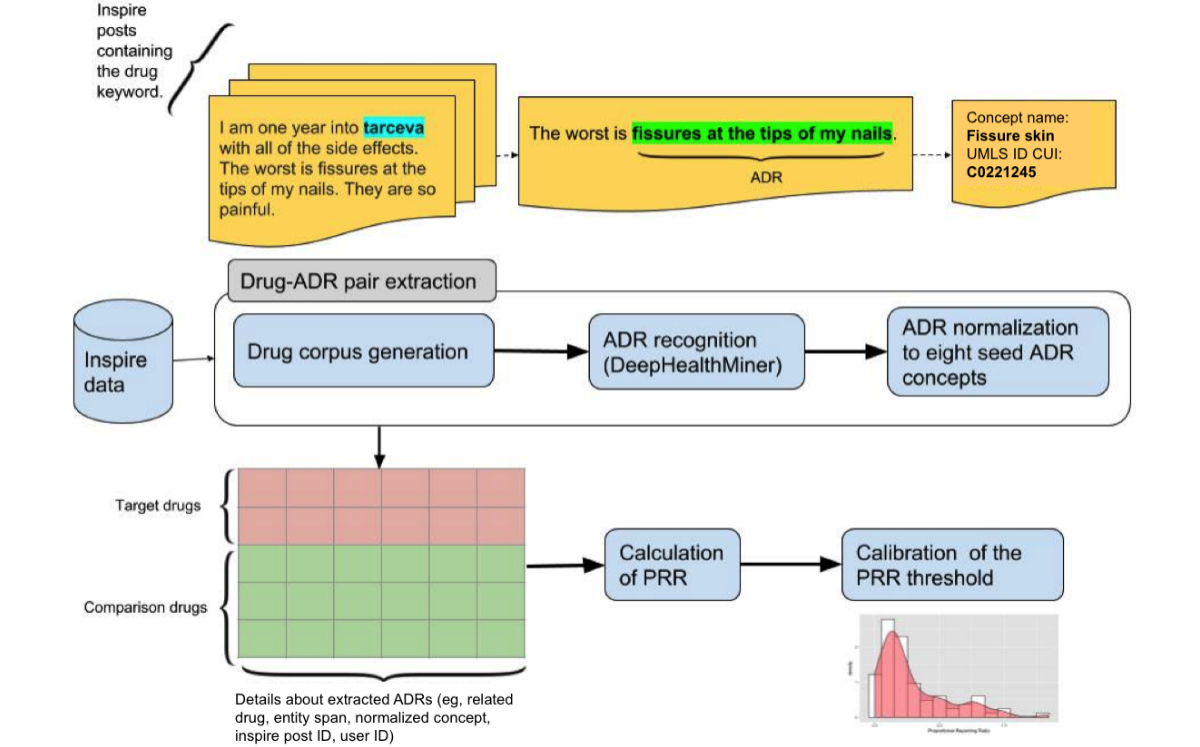
Pipeline to identify adverse drug reaction (ADR) signals associated with epidermal growth factor receptor (EGFR) and programmed cell death 1 (PD-1) inhibitors in social health networks. For drug-ADR pair extraction, for each drug, we generate a collection of user posts containing at least one mention of the drug. This drug corpus is then processed via DeepHealthMiner to recognize mentions of ADRs. The extracted mentions are then mapped to the corresponding Unified Medical Language System (UMLS) Concept Unique Identifiers (CUIs). The identified drug-ADR pairs and the related details, for both target and comparison group, are then stored in a relational database. The proportional reporting ratio (PRR) is calculated to quantify the drug-ADR relations. We calibrated the score using the distribution of the negative control set.

Figure 2 depicts our overall pipeline and graphical depiction of the above methods.

## Results

### Finding Drug-Adverse Drug Reaction Signals From Forum Posts

We identified 50,574 Inspire posts related to EGFR inhibitors and 16,598 related to checkpoint inhibitors; a total number of 13,600 ADR concepts extracted from the former and 812 concepts from the latter mapped to the eight target ADRs. We evaluated the performance of the presented NER method in identifying ADR mentions from the Inspire test set and achieved a microaveraged *F*-measure of 0.738 (0.731 precision and 0.745 recall).

To assess system performance in extraction and normalization of ADR concepts, we performed additional manual validation of the extracted ADR concepts as described in the Methods. Our final validation set for the entire pipeline for identifying drug-ADR pairs consisted of an additional 120 posts which had 15 randomly selected posts for every ADR that the system extracted for one of our target drugs.

We considered an extracted concept as a true positive if the system correctly detected the relevant span of text; classified the entity as an ADR rather than indication; and normalized the ADR to the correct concept. Benchmarking results show that our system achieved a high performance (microaverage precision of 0.90) in recognizing and normalizing the ADRs in the user posts.

The distribution of the frequencies and the calculated PRR of the skin ADRs extracted from the EGFR inhibitor-related posts correlates closely with published observed rates in patients (see Figure 1), further validating the pipeline for identifying ADRs. The PD-1 inhibitor ADR landscape is largely limited to case reports; forum frequencies and PRR values correlate with published rates, suggesting that those described to date accurately represent the emerging toxicity landscape (see Figure 1).

**Figure 3:**
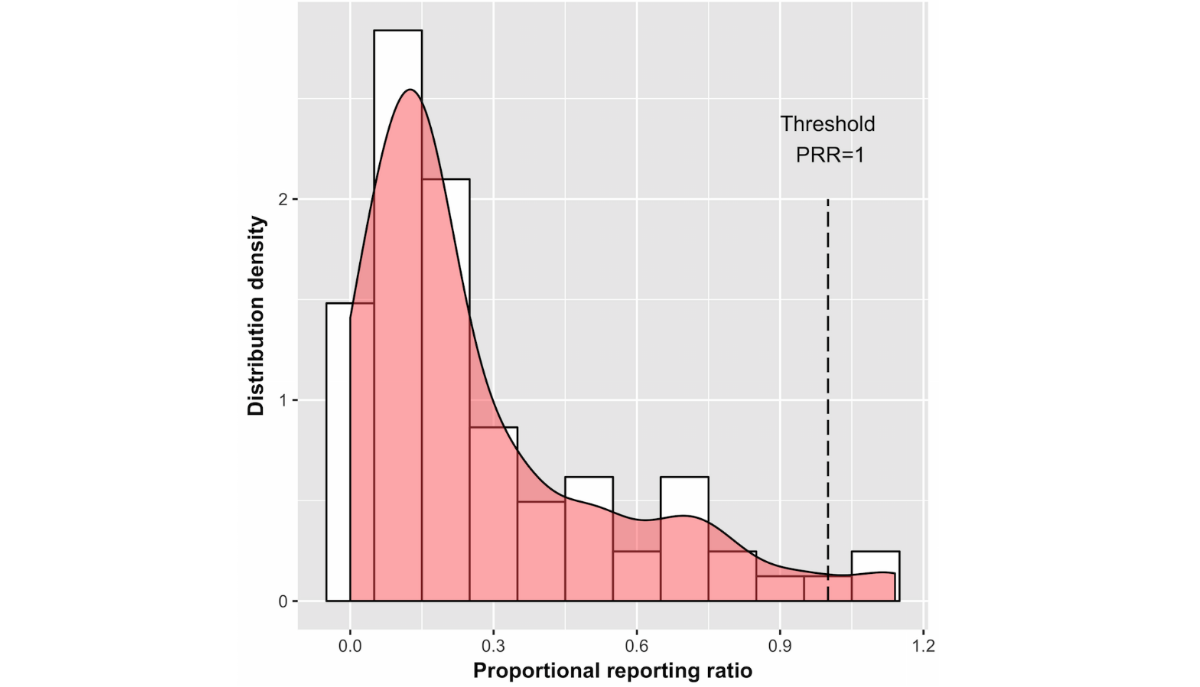
Proportional reporting ratio (PRR) distribution for a set of 28 negative example drugs representing 81 drug-adverse drug reaction (ADR) pairs. The mean is 0.12, median is 0.2, and maximum is 1.4, highlighting a PRR threshold of 1, below which <5% of drug-ADR pairs have true associations.

To determine the threshold at which PRR represents true signal warranting further investigation, we plotted the PRR distribution for the 81 drug-ADR pairs in the negative control set (see Figure 3). More than 95% of the pairs in this set have a PRR of less than 0.82; therefore, drug-ADR pairs with a PRR greater than 1 can be considered as signaling true ADRs.

### Adverse Drug Reactions are Described in Internet Forums Prior to Published Reports

**Figure 4:**
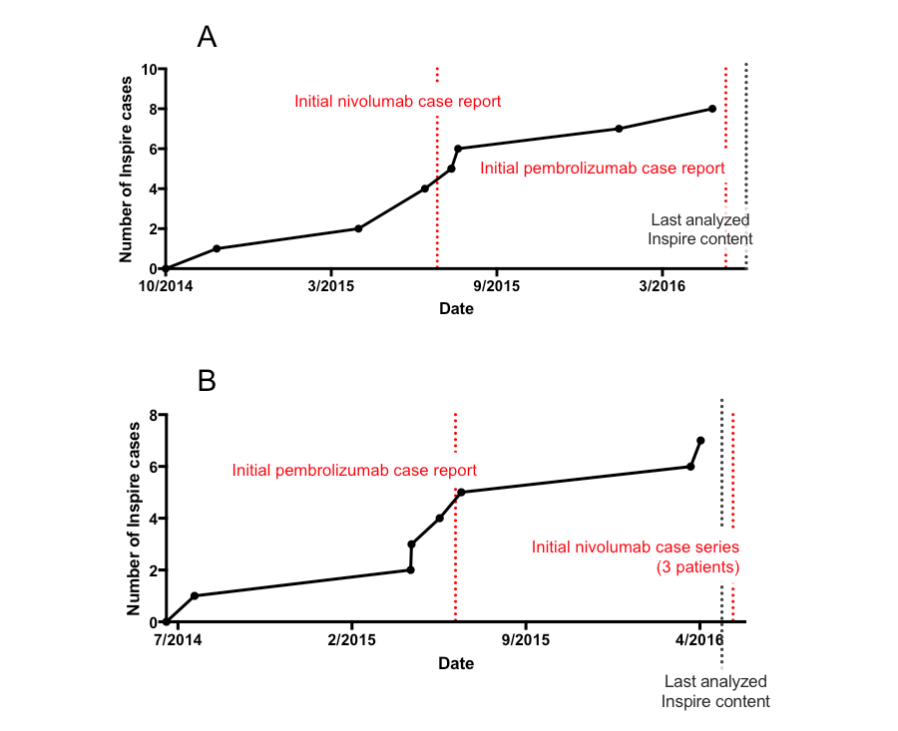
Cutaneous adverse drug reactions (ADRs) identified in Inspire forums precede initial published clinical reports. We plotted cumulative post count (y-axis) at each date (x-axis) for time-to-detection analysis. (A) Psoriasis was first reported in the literature as individual case reports with programmed cell death 1 (PD-1) inhibitors in July 2015 and May 2016. Inspire users began describing psoriasis flares 9 months prior to the first case report. (B) Bullous reactions with checkpoint inhibitors were first reported in the literature as a case report with pembrolizumab in May 2015 and as a three-case series with nivolumab in May 2016. Inspire cases were reported online 9 months before the initial case report.

To compare the timing of online ADR reports to initial ADR reports in the literature, we looked at both common and rare events associated with our target drugs. Papulopustular (ie, acneiform) rash and fingernail changes are well-reported ADRs associated with erlotinib and were first described in published case reports in September 2005 [41] and September 2006 [42], respectively. Inspire posts for these reactions appeared 5 and 3 months in advance of publication, respectively. Psoriasis in the setting of PD-1 inhibitor treatment was first documented in case reports in July 2015 [43] and May 2016 [44]. Inspire forum posts describing this ADR preceded the 2015 case report by 9 months (see Figure 4A). Blistering reactions with PD-1 inhibitors were initially published as a case report in June 2015 [45] and as a three-case series in May 2016 [46]. Forum descriptions preceded the first case report by 9 months (see Figure 4B). Taken together, our data suggest a significant and consistent reporting lead-time advantage in online health forum posts. Cutaneous ADRs in both EGFR and PD-1 inhibitors have been reported to be associated with cancer response to therapy [47-50], highlighting the clinical utility of early detection.

### Novel Adverse Drug Reaction Discovery in Social Health Networks

In early 2017, we documented three patients on erlotinib reporting hypohidrosis. Our system detected 23 unique Inspire users reporting hypohidrosis in causal association with erlotinib as early as 2006, based on manual review of user posts, with a PRR score of 1.90 (see Figure 3). This predicted ADR is absent from over 15 years of EGFR inhibitor literature, implicating a novel ADR detected through our system extractions from online posts. EGFR is expressed in sweat glands [51] and decreases with pharmacologic EGFR inhibition [52]. Interestingly, the hypohidrotic ectodermal dysplasia phenotype is partially mediated by decreased EGFR signaling [53], which can possibly cause hypohidrosis. These findings demonstrate the potential of ADR extraction from Internet forums to not only detect ADRs earlier, but also to contribute to novel ADR discovery.

## Discussion

Our retrospective analyses demonstrate the potential of conducting targeted ADR surveillance in real time. Because many chemotherapeutics have nonspecific side-effect profiles, including nausea, vomiting, and fatigue, which are shared with most antineoplastics as well as thousands of other drugs, we see greater relevance in seeking specific signal-to-noise ratios based on clinically generated hypotheses—that is, reactions seen with a drug, with a hypothesized causal association. For example, our impetus for mining for hypohidrosis in association with erlotinib stemmed from a single clinical encounter. As we built and applied our pipeline to the Inspire dataset, we eventually saw two more patients in clinic with the same symptom; together, with the 23 Inspire users we identified, our system reported statistically significant association, supporting the classification of this symptom as a true ADR.

The presented methods in the signal generation pipeline can easily be applied to other drugs and ADRs. Our NER system extracts ADRs independent of a specific drug and, therefore, the system performance should easily translate to the study of other drugs. In fact, in our previous studies [17,29], we showed that the presented NER methods tested on a dataset of user posts about 81 different drugs achieved high performance in extracting mentions of ADRs, not limited to any specific ADR. Therefore, the NER component can easily be applied to the majority of ADRs. However, if the goal is to identify specific rare ADRs or mentions that often are presented in contexts different than ADRs (eg, psoriasis), the system may be improved by adding additional relevant human-labeled training examples as we did in this study. Future research is needed to further explore transfer learning techniques for improvement in the performance of NER models when applied to the new tasks.

Furthermore, with respect to other ADRs, the only additional effort needed for their inclusion would be normalization and determination of grouping of the UMLS concepts attributed to a single ADR (eg, the concepts that can be considered as “nail changes”). For normalization, we used the seed ADRs to identify and group the concepts that indicated the target ADRs in our study. Overall, the precise normalization of medical mentions in text is a challenging task in biomedical information extraction [36]; particularly for the medical entities in user posts [54], future research could explore alternative techniques for optimal normalization of medical concepts in social media postings.

Our study demonstrates that it is possible to detect ADR signals from patient-generated social media posts an average of 7 months earlier than literature reporting and at frequencies comparable to their eventual literature descriptions. We describe the proof-of-principle construction and validation of a signal generation pipeline for ADR detection from social health networks. We benchmark our system extractions of known drug events against their literature reports to evaluate our pipeline’s accuracy and temporal advantage. Our system is able to extract drug-ADR signals from highly unstructured online patient content with high precision.

In this work, we demonstrate the utility of mining online patient reports to identify signals for both common and rare ADRs with high precision. We envision the potential for social media-based signals to be combined with those derived from alternative modes—electronic health records, insurance claims, and FDA reports—to construct the most comprehensive and dynamic catalogue of ADRs.

Looking ahead, we envision the utility of a live pharmacovigilance system that reports emerging drug-ADR association signals over the course of days to weeks and sends these reports to physicians for review and comparison with clinical experience. This would enable tracking of individual reports and changing PRRs over time, providing the reviewing physicians with access to a significantly broader patient cohort than seen in single treatment centers. We do anticipate potential new challenges in capturing how patients describe ADRs that are not directly visible to them, which will be an engaging and important area of further study.

If a reporting system such as ours were extended to public use, we also anticipate the inherent risks and challenges associated with patient access. While many patients search for their health concerns before presenting to clinicians, they are often misguided, which may impact the decision to seek care or timing of visit to a physician. A system such as ours, which captures the experiences of millions of patients, may be able to offer context for patients to understand their concerns, but it would be a further challenge to offer triaging of ADR severity to patients based solely on the reports of other patients and should instead serve to motivate patients to discuss their concerns in a timely manner with their own doctors.

## Appendix

Multimedia Appendix 1 List of drug names.

Multimedia Appendix 2 List of seed adverse drug reaction (ADR) concepts used.

Multimedia Appendix 3 List of comparison drugs.

Multimedia Appendix 4 List of negative control drug-adverse drug reaction (ADR) pairs.

## Abbreviations

ADR: adverse drug reaction
CUI: Concept Unique Identifier
DHM: DeepHealthMiner
EGFR: epidermal growth factor receptor
FDA: US Food and Drug Administration
NER: named entity recognition
PCOS: polycystic ovary syndrome
PD-1: programmed cell death 1
PRR: proportional reporting ratio
SIDER: side-effect resource
SRS: spontaneous reporting system
UMLS: Unified Medical Language System
