# Supplementary material for: Early Detection of Adverse Drug Reactions in Social Health Networks: A Natural Language Processing Pipeline for Signal Detection
Source: JMIR Public Health Surveill. 2019 Jun 3;5(2):e11264. doi: 10.2196/11264 (PMC6684218; doi:10.2196/11264)
Supplement: Multimedia Appendix 1 [file publichealth_v5i2e11264_app1.pdf]

<table>
<tr><th>Drug</th><th>Drug_Group</th><th>MYSQL_Regex</th><th>Comment</th></tr>
<tr><td>nivolumab,nivo,keytruda,pembro,opdivo,pd1</td><td>Immune checkpoint inhibitors</td><td>" body regexp '[[:<:]]"+drugName+".*' "</td><td>We designed a regex specific to pd1 related mentions that identfies mentions like pd-1, pd_l1 or anti-PD-1 and other alternatives: ('[[:<:]](anti[_|-|])?pd.{0,3}[1][[:>:]]')</td></tr>
<tr><td>tarceva,erlotinib,cetuximab,erbitux,gefitinib,iressa,lapatinib,tykerb,panitumumab,vectibix,caprelsa,vandetanib,portrazza,necitumumab,tagrisso,osimertinib</td><td>EGFR inhibitors</td><td>" body regexp '[[:<:]]"+drugName+".*' "</td><td></td></tr>
</table>
