# Supplementary material for: Early Detection of Adverse Drug Reactions in Social Health Networks: A Natural Language Processing Pipeline for Signal Detection
Source: JMIR Public Health Surveill. 2019 Jun 3;5(2):e11264. doi: 10.2196/11264 (PMC6684218; doi:10.2196/11264)
Supplement: Multimedia Appendix 2 [file publichealth_v5i2e11264_app2.pdf]

Seed ADR concepts and commonly used colloquial phrases.

ADR	Seed UMLS Concepts	Common colloquial phrases
Rash or acne	Rashes, eruptions and exanthems NEC: C0947945<br>Rash: C0015230<br>Rash generalized: C0497365<br>Rash macular: C0221201<br>Rash maculo-papular: C0423791<br>Rash papular: C1519353<br>Rash trunk: C0241502<br>Rash face: C0239521	
Acne	Acne: C0702166<br>Pustular conditions: C0851794<br>Rash pustular: C0085641<br>Rash acneiform: C0175167<br>Acne vulgaris: C0001144<br>Acne NOS: C0702166	
Pruritus, itchy skin	Pruritus: C0033774<br>Pruritus NEC: C0876975<br>Rash pruritic: C0033771<br>Itchy skin: C0033774<br>Itchy rash: C0033771<br>Itching – generalised: C0475858	
Paronychia, nail changes	Nail finding: C0423800<br>Abnormality of nail of toe: C3839753<br>Change in nail texture: C0277953<br>Disorder of nail: C0027339<br>Finding of appearance of nail: C0574755<br>Finding of nail growth: C0574758<br>Nail bed finding: C0423839<br>Nail fold finding: C0423836<br>Nail changes: C0234909<br>Paronychia: C0030578<br>Paronychia of fingers of bilateral hands: C2034557<br>Paronychia of finger: C0311210<br>Paronychia of toe: C0311211<br>Fissure in skin: C0221245	nail fissure, nail fall off
Xerosis, dry skin	Xerosis: C0259817<br>Skin xerosis: C0263465<br>Dry skin: C0151908<br>Dry skin aggravated: C1739408	dry out skin, dry out scalp, dry out finger, dry out face, dry face, skin driness, dry out hand
Hypohidrosis	Disorder of sweat gland: C0038986<br>Anhidrosis: C0003028<br>Apocrine and eccrine gland disorders: C0852354<br>Hypohidrosis: C0020620<br>heat intolerance: C0231274	over heat, dry out, unable perspire, unable sweat, difficult perspire, difficult perspiration, hard perspiration, hard

	heat sensitive:C0231274<br>overheat:C3276621<br>perspire: C0038984 (negated)<br>sweat: C0038984 (negated)	perspire, decrease perspiration, decrease sweat, stop perspire, stop perspiring, inability perspire, barely sweat
Bullous eruption, blister	Bullous lesions: C0005758<br>Dermatitis bullous: C0085932<br>Bullous pemphigoid NOS: C0030805<br>Rash pemphigoid: C0542175<br>Pemphigoid reaction: C0235853<br>Pemphigus-like lesion: C0860539<br>Vesicular rash: C0221203<br>Bullous pemphigoid: C0030805<br>Vesicular eruption: C0263237<br>Drug eruption vesicular: C0858702<br>Blistering: C0005758<br>Blistering of mouth: C0587288<br>Rash bullous: C0235819<br>Immunobullous disease: C1274166<br>Blistering eruption: C0344311<br>Blister of skin without infection: C1302857<br>Bullous dermatosis: C0085932	
Psoriasis	Psoriasis: C0033860<br>Psoriatic arthropathy: C0003872<br>Plaque psoriasis: C0406317<br>Skin plaque: C0241148<br>Psoriatic conditions: C1697846	
