# Supplementary material for: Early Detection of Adverse Drug Reactions in Social Health Networks: A Natural Language Processing Pipeline for Signal Detection
Source: JMIR Public Health Surveill. 2019 Jun 3;5(2):e11264. doi: 10.2196/11264 (PMC6684218; doi:10.2196/11264)
Supplement: Multimedia Appendix 3 [file publichealth_v5i2e11264_app3.pdf]

xeloda
taxotere
avastin
forteo
thyrogen
cytomel
domperidone
gabapentin,neurontin
zometa
lyrica
follistim
tamoxifen
reglan
reclast
metformin
fosamax
plavix
letrozole
herceptin
levothyroxine,synthroid
alimta
cisplatin
lupron
doxil
gemzar
clomid
cytoxan
