# Supplementary material for: Early Detection of Adverse Drug Reactions in Social Health Networks: A Natural Language Processing Pipeline for Signal Detection
Source: JMIR Public Health Surveill. 2019 Jun 3;5(2):e11264. doi: 10.2196/11264 (PMC6684218; doi:10.2196/11264)
Supplement: Multimedia Appendix 4 [file publichealth_v5i2e11264_app4.pdf]

**Drug**	**Condition**
herceptin	hypohidrosis
herceptin	psoriasis
xeloda	acne
xeloda	psoriasis
taxotere	psoriasis
avastin	itch
avastin	hypohidrosis
avastin	psoriasis
forteo	acne
forteo	itch
forteo	nail problems
forteo	dry-skin
forteo	hypohidrosis
forteo	blister
forteo	psoriasis
thyrogen	acne
thyrogen	nail problems
thyrogen	hypohidrosis
thyrogen	blister
thyrogen	psoriasis
cytomel	rash
cytomel	acne
cytomel	hypohidrosis
cytomel	blister
cytomel	psoriasis
domperidone	rash
domperidone	acne
domperidone	nail problems
domperidone	dry-skin
domperidone	hypohidrosis
domperidone	blister
domperidone	psoriasis
zometa	psoriasis
lyrica	acne
follistim	nail problems
follistim	blister
follistim	psoriasis
tamoxifen	acne
tamoxifen	hypohidrosis
reglan	acne
reglan	nail problems
reglan	dry-skin
reglan	hypohidrosis
reglan	blister
reglan	psoriasis
reclast	acne
reclast	nail problems
reclast	dry-skin
reclast	hypohidrosis
reclast	blister
reclast	psoriasis
metformin	dry-skin

metformin	blister
fosamax	acne
fosamax	nail problems
fosamax	hypohidrosis
fosamax	blister
plavix	acne
plavix	nail problems
plavix	dry-skin
plavix	hypohidrosis
plavix	psoriasis
letrozole	acne
letrozole	nail problems
letrozole	hypohidrosis
levothyroxine	nail problems
levothyroxine	blister
alimta	blister
alimta	psoriasis
cisplatin	psoriasis
lupron	hypohidrosis
lupron	psoriasis
doxil	acne
gemzar	psoriasis
clomid	nail problems
clomid	dry-skin
clomid	blister
clomid	psoriasis
cytoxan	dry-skin
cytoxan	hypohidrosis
cytoxan	psoriasis
